# Supplementary material for: Metal Exposure-Related Welder’s Pneumoconiosis and Lung Function: A Cross-Sectional Study in a Container Factory of China
Source: Int J Environ Res Public Health. 2022 Dec 14;19(24):16809. doi: 10.3390/ijerph192416809 (PMC9779211; doi:10.3390/ijerph192416809)
Supplement: Supplementary file 1 [file ijerph-19-16809-s001.zip › ijerph-1981536-supplementary.pdf]

Table S1. The pneumoconiosis stages and disease progression of 21 cases.

Case No.	Stage of disease (first diagnosis)	Stage of disease (last diagnosis)	Time span (years)	Variation of disease stage
1	I	I	9	stable
2	under medical surveillance	I	8	aggravated
3	I	I	9	stable
4	I	I	9	stable
5	I	II	9	aggravated
6	I	I	9	stable
7	II	II	8	stable
8	I	II	9	aggravated
9	I	II	10	aggravated
10	I	II	10	aggravated
11	I	II	10	aggravated
12	I	I	10	stable
13	I	I	10	stable
14	II	II	10	stable
15	II	II	10	stable
16	II	II	10	stable
17	II	II	10	stable
18	II	II	10	stable
19	II	II	10	stable
20	II	II	10	stable
21	II	II	10	stable

"Under medical surveillance" means when health examination reveals indeterminate pneumoconiosis-like imaging changes in X-ray chest radiographs, subjects would be required to leave dust-exposure occupational environment and undergo dynamic observation within a certain period of time.

Table S2. LOD and LOQ for metal content detection.

Metal	LOD (μg/L)	LOQ (μg/L)
Cr	0.05	0.16
Mn	0.12	0.40
Co	0.02	0.06
Ni	0.12	0.40
Cu	0.18	0.61
Zn	0.13	0.44
Mo	0.02	0.08
Cd	0.01	0.03
Pb	0.04	0.12
Fe	9.00	27.00

***Abbreviations*:** LOD, limits of detection; LOQ, limits of quantitation.

Table S3. Chemical composition (%) of welding wires.

Items	Requirement	Test Results
C	0.060 - 0.150	0.073
S	≤ 0.025	0.011
Mn	1.40 - 1.85	1.42
Si	0.80 - 1.15	0.85
P	≤ 0.025	0.012
Cr	≤ 0.150	0.023
Ni	≤ 0.150	0.012
Mo	≤ 0.150	0.006
V	≤ 0.030	0.003
Cu	≤ 0.500	0.114

Table S4. Comparison of haemal and urinary metal concentrations of welders in each subgroup.

	Manual welding (n=149)	Automatic welding_low exposure (n=113)	Automatic welding_high exposure (n=29)	*p*
Element content in blood				
Sample size	149	113	29	
Cr	2.73 (0.53)	2.83 (0.68) [a]	3.18 (0.29) [a,b]	<0.001
Mn	4.70 ± 0.52	4.70 ± 0.59	4.89 ± 0.38	0.191
Co	0.59 (0.44)	0.47 (0.47)	0.79 (0.33) [a,b]	0.004
Ni	2.76 (0.82)	2.70 (1.01)	3.10 (0.36) [a,b]	<0.001
Cu	9.75 ± 0.16	9.69 ± 0.17 [a]	9.75 ± 0.12	0.003
Zn	12.47 ± 0.28	12.55 ± 0.31	12.48 ± 0.26	0.077
Mo	1.49 (0.50)	1.43 (0.53)	1.59 (0.47) [b]	0.03
Cd	1.41 (1.26)	1.85 (1.31) [a]	1.45 (1.30)	0.028
Pb	5.01 (0.51)	5.09 (0.54)	5.16 (0.51)	0.22
Fe	9.05 ± 0.17	9.13 ± 0.15 [a]	9.03 ± 0.16 [b]	<0.001
Element content in urine				
Sample size	142	108	28	
Cr	1.18 (1.20)	1.57 (0.98)	1.10 (0.61) [b]	0.005
Mn	2.19 ± 0.80	2.28 ± 0.77	1.89 ± 0.74	0.07
Co	0.52 (0.50)	0.48 (0.30)	0.57 (0.35)	0.37
Ni	3.04 ± 0.80	2.89 ± 0.74	2.93 ±0.68	0.335
Cu	7.08 (0.67)	7.11 (0.77)	7.23 (0.49)	0.056
Zn	8.76 ± 0.87	8.52 ± 1.11	8.17 ± 0.75 [a]	0.007
Mo	6.16 (1.24)	5.92 (1.40)	5.93 (1.85)	0.144
Cd	0.86 (0.46)	0.91 (0.54)	0.67 (0.59)	0.142
Pb	1.20 (0.90)	1.06 (0.87)	0.88 (0.53) [a]	0.033
Fe	8.93 ± 0.95	8.46 ± 1.07 [a]	8.39 ± 1.20 [a]	<0.001

Values are mean ± SD, median (IQR), or number (%). [a] Compared with manual welding by Dunn's test or LSD t-test. [b] Comparison between automatic welding-low exposure and automatic welding-high exposure group by Dunn's test or LSD t-test. $P < 0.05$ indicates statistical significance.

Figure S1. Sketch of welding workplace and sampling sites.

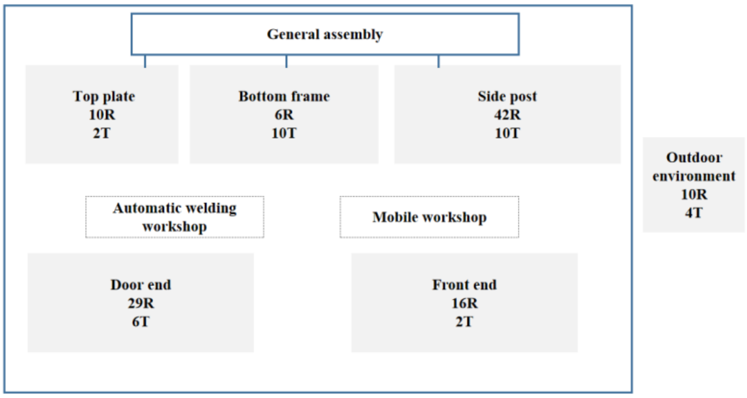

Container production is an assembly line, and different workshops are responsible for different welding tasks. The front-end and door-end workshops are mainly responsible for assembling and welding the front and rear doors and side panels of the container. The general assembly workshop is responsible for welding the bottom frame, the four-sided panels, and the top plate into finished products. R indicates respirable dust sampled by personal sampling method. T indicates total dust sampled by area sampling method. We collected different numbers of samples in each workshop.

Supplemental material methods

The spirometer we used, MasterScreen portable spirometer (Jaeger, Germany), is a portable spirometer with a laminar pressure differential flow sensor. The system has a built-in estimated value system jointly developed by Peking Union Medical College Hospital, Chaoyang Hospital, and Shanghai Zhongshan Hospital, providing a reference standard for lung function of the Chinese population. This device has been used to measure lung function in several large-scale cohort studies in China over the past 10 years. Two trained physicians performed spirometry. Before the test, subjects took a seat to stabilize their breaths. They were required to measure lung function at least 3 times and the optimal values were used for analysis. The specific standard procedures and methods for spirometry are as follows:

1 Quality control before testing

1.1 Calibration of environmental parameters: Use the electronic weather instrument to determine the room temperature, relative humidity, and atmospheric pressure. Input them into the environmental parameter module.

1.2 Standard flow rate-capacity calibration: Connect the elbow, filter, and silicone connector of the sensor to the 3L calibration cylinder, and calibrate at least once a day.

1.3 Confirm subjects' information: Explain and demonstrate the measurement procedures to subjects. Avoid drinking alcohol within 4 hours, eating large amounts of food within 2 hours, smoking within 60 minutes, and strenuous exercising within 30 minutes before the measurement.

2 Quality control during testing

2.1 Reliability assessment: Taking the flow rate-volume curve as a reference, subjects inhale to the total lung volume. The initial period of forced exhalation: first, it must be confirmed whether the initial period of forced exhalation is exhausted (extrapolated volume < 0.15L, expiratory time ≥ 6s or end-expiratory plateau ≥ 2s). Observe the flow rate-volume curve, and a steep ascending branch of the respiratory phase and a smooth descending branch are required. A closed curve between the inspiratory and expiratory phases is needed. Avoid coughing and glottis closure.

2.2 Repeatability evaluation: each subject must repeat the measurement more than 3 times, and at least 3 times test results meet the reliability evaluation. The software will automatically compare and evaluate the three measurement results, requiring that the difference between the maximum and minimum FVC be less than 0.15L or 5% of FVC, and the difference between the maximum and the minimum PEF be less than 0.67L/s.
